# In recent research: exosomal and molecular regulators of browning and thermogenesis in adipose tissues

**DOI:** 10.55730/1300-0144.6093

**Published:** 2025-10-03

**Authors:** Merve İNEL, Bahadır ÖZTÜRK

**Affiliations:** 1 Department of Medical Biochemistry, Institute of Health Sciences, Selçuk University, Konya, Turkiye; 2 Department of Medical Biochemistry, School of Medicine, Selçuk University, Konya, Turkiye

**Keywords:** Brown adipose tissue, beige adipose tissue, transcriptional factors, exosome, miRNA

## Abstract

This review synthesizes current knowledge on the molecular mechanisms regulating brown, white, and beige adipose tissues, with particular emphasis on the browning process of white adipose tissue. Rather than considering adipose tissue as a passive energy reservoir, this review underscores its active role as a dynamic endocrine and metabolic organ. Brown and beige adipose tissues, recognized for their thermogenic capacity and contribution to energy expenditure, have emerged as promising therapeutic targets for the treatment of obesity, type 2 diabetes, and related metabolic disorders. The review draws upon recent findings on adrenergic signaling, transcription factors, exosomal microRNAs, and other novel regulatory pathways for the creation of a cohesive framework. Particular attention is paid to how environmental, physiological, and molecular cues, such as exposure to cold, exercise, gut microbiota, and exosomal communications, converge on common pathways to influence thermogenesis and browning. By highlighting these interrelated mechanisms, the review not only summarizes recent advances in the field, but also clarifies their interconnected implications for metabolic regulation and potential therapeutic interventions.

## 1. Introduction

The global obesity epidemic has intensified the search for new therapeutic strategies to combat weight gain, type 2 diabetes and metabolic dysfunction [1,2]. Chronic inflammation in organs such as the liver, brain, pancreas, and adipose tissue can disrupt tissue balance and cause metabolic diseases [3]. Adipose tissue is now recognized as an active endocrine organ rather than a mere inert calorie depot. It modulates appetite, insulin sensitivity, inflammatory processes, and thermal regulation through the secretion of adipokines, thereby coordinating metabolic cross-talk with other organs [4–6].

White adipose tissue (WAT), while essential for energy storage, contributes to inflammation and insulin resistance when present in excess, particularly in the visceral depot. In contrast, beige adipose tissue (BeAT) functions as a metabolic “switchboard”, acquiring brown-like thermogenic properties under certain stimuli, thus counterbalancing the detrimental effects of WAT expansion. Brown adipose tissue (BAT), with its high mitochondrial density and thermogenic capacity, further exemplifies the therapeutic promise of adipose biology in metabolic disease [7–9].

**Figure 1:**
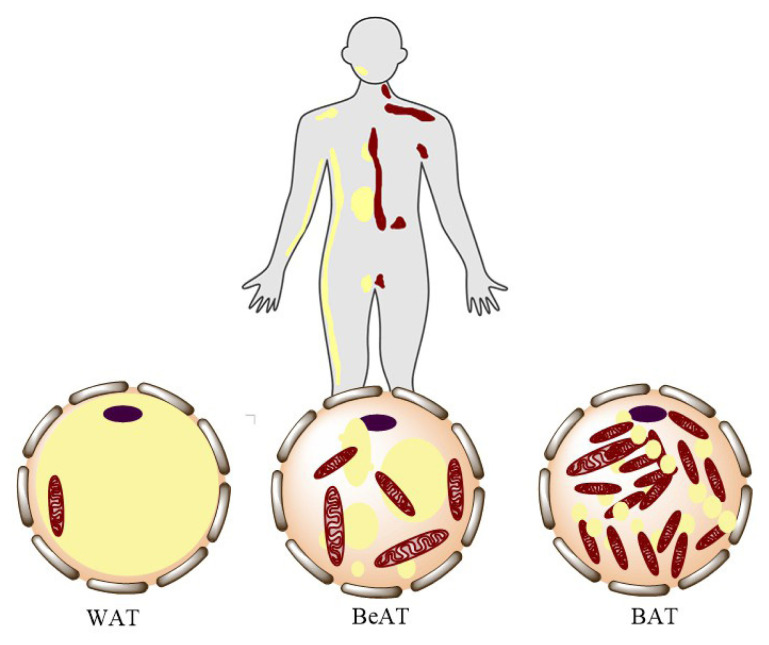
Adipose tissues and regions in the body.

A unifying theme in adipose research is the heterogeneity and location-specific function of adipose depots. WAT is broadly distributed, especially in subcutaneous and visceral locations, and specializes in triglyceride storage and fatty acid release, as shown in Figure 1. Excess visceral WAT uniquely correlates with cardiometabolic risk, while subcutaneous depots can harbor beige adipocytes capable of thermogenesis. While BAT was initially identified in the 16th century, its significance in adults has only recently been appreciated, persisting mainly in cervical and supraclavicular regions in adults, but in the intrascapular region of rodents and neonates [10–17].

At a cellular level, the defining feature of BAT is its abundance of mitochondria and multilocular lipid droplets that enable adaptive thermogenesis via uncoupling protein 1 (UCP1). By dissipating fuel energy as heat rather than storing it as ATP, UCP1-mediated non-shivering thermogenesis distinguishes BAT from muscle-driven heat production. Beyond the acute thermogenic responses to cold, BAT also contributes to glucose and lipid metabolism, cardiovascular health, and body weight regulation [18–23]. Notably, BAT activity varies from person to person, correlating inversely with body mass index and fat percentage. This variability underscores the therapeutic potential of augmenting BAT function or inducing WAT browning for the treatment of obesity and diabetes.

The early 2000s marked the discovery of beige adipocytes – intermediate, “convertible” cells within WAT depots that acquire BAT-like traits under adrenergic or nutritional stimuli [25–30]. This phenomenon of “browning” represents a plastic adaptation of adipose tissue and is a key focus of this review, given its potential to harness adipocyte diversity for metabolic benefit.

## 2. Browning and BAT activity regulators

In this section, browning and BAT activation are identified as an outcome of overlapping environmental, nutritional, and physiological cues acting through shared molecular pathways, rather than as isolated phenomena. Repeated exposure to cold, for instance, reliably activates BAT and stimulates the browning of WAT in humans [26]. This effect reflects the involvement of diverse external stimuli, including temperature fluctuations to dietary components, ultimately converging on β-adrenergic signaling, UCP1 activation, and mitochondrial thermogenesis.

Cold exposure increases sympathetic activity, leading to norepinephrine release and the subsequent activation of β-adrenergic receptors, which directly stimulate UCP1 expression [31].

Certain stimuli such as diet, exercise and pharmacological agents can trigger beiging [26,28,32,33–35]. Dietary factors reinforce this axis. For example, short-chain fatty acids generated through gut microbial fermentation, as well as nutrients like omega-3 fatty acids, capsaicin, and resveratrol, enhance BAT thermogenesis, in part by modulating insulin sensitivity, mitochondrial activity, and transcriptional regulators such as PPARγ. [31,36–38]. Some nutrients, such as ginger, green tea, cinnamon, and coffee have demonstrated anti-obesity effects, increasing BAT activity by supporting the development of BeAT [39–41]. In short, these nutritional inputs integrate microbial, endocrine, and cellular networks to shape adipose tissue behavior.

In both invitro and in vivo studies, stem cell-derived adipocytes exposed to coffee increased oxygen consumption and brought about structural changes in mitochondria, evidenced by elevated UCP1 protein expression and metabolic activity. These effects were associated with delayed body mass gain and improved fat distribution in obese mice, and increased temperatures in the supraclavicular region in adult humans [42,43]. The identification of thermogenic dietary factors can provide a broader perspective, and can steer nutritional intervention strategies for the prevention and management of obesity and diabetes. However, the nutritional modulation of adaptive thermogenesis just one part of a complex puzzle that requires multidisciplinary studies and evaluations for the creation of viable treatment strategies.

Exercise can serve as an additional systemic input to the browning program, releasing myokines such as irisin that act as “messengers” between muscle and adipose. Compounds that mimic exercise, such as AMPK agonists, highlight how pharmacological interventions can tap into the body’s natural biological pathways [44–46]. Gut microbiota add another regulatory layer, with microbial metabolites such as bile acids triggering receptors like TGR5 that fine-tune BAT thermogenesis [47–52]. In a study examining the potential transmission of BAT activity through the microbiome, fecal transplantation in mice had no effect on BAT activity [24]. It has been shown that interventions with prebiotics and probiotics regulate adaptive thermogenesis and browning by affecting the composition of the gut microbiome, while prebiotic support following antibiotic use upregulates the amount of BAT and supports its activity [53–56]. These results have contributed to the development of safe and effective interventions that can stimulate BeAT formation and increase BAT activity.

## 3. Regulators of thermogenesis at a molecular level

Just as external signals interact to control adipose function, intracellular regulation is achieved through an intricate interplay of receptors and transcriptional factors.

### 3.1. Adrenergic receptors

UCP1 and UCP3 are members of the uncoupling protein family with links to energy homeostasis. UCP1 is the dominant effector of non-shivering thermogenesis in BAT, whereas UCP3 appears to play more context-dependent roles. BAT is a metabolically active tissue that dissipates energy as heat mainly through UCP1. In previous studies, UCP1 has been recognized as the primary mediator of thermogenesis in brown adipose tissue, based on findings indicating that mice lacking UCP1 are unable to efficiently generate heat, and subsequently develop hypothermia under cold conditions. A creatine-dependent substrate cycling pathway that complements UCP1-driven heat production has been identified, known as creatine kinase B (CKB). As the most abundant isoenzyme in brown adipocytes, and the only one found in cultured primary brown adipocytes, CKB co-localizes with UCP1 and supports cold-induced energy expenditure in thermogenic adipocytes [57]. The above findings indicate that BAT thermogenesis arises from both UCP1-centric and UCP1-independent (creatine cycle) mechanisms, which are able to operate in parallel.

The primary physiological function of BAT is to respond to β-adrenergic signals through the induction of thermogenesis. In brown adipocytes, β-adrenergic receptor activation elevates cyclic AMP and activates protein kinase A, which phosphorylates the targets that promote lipolysis and mitochondrial respiration, thereby enabling and amplifying UCP1 activity [11]. In β-adrenergic stimulation, three subtypes of the adrenergic receptor can be found: β1 (ADRB1), β2 (ADRB2), and β3 (ADRB3), and receptor subtype usage differs from species to species. Among these signals, ADRB1 in particular is the dominant adrenergic receptor in human BAT [58]. Although ADRB3 activity has been proposed to contribute to the maintenance of overall BAT health [59], expression profiling shows a human-specific pattern. In a study examining the activation of these receptors, ADRB1 was found to be expressed most in BAT when compared to WAT, while ADRB3 was the least expressed [60]. In a study reporting similar results, ADRB3 was identified as active in rodent brown fat activation, but was not detected in human WAT [61]. This rodent-human divergence likely underlies the limited clinical efficacy of ADRB3 agonists for the treatment of obesity in humans, and shifts therapeutic emphasis toward modulating ADRB1-driven pathways in human BAT [60].

### 3.2. Transcriptional factors

**Figure 2:**
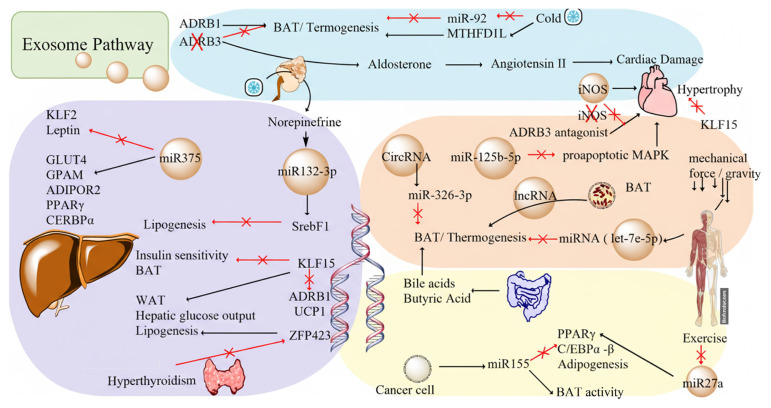
Mechanism of action of adipose tissue metabolism factors.

Although there have been few studies to date mapping the adipose transcriptional circuitry, converging evidence reveals a coordinated network of activators and repressors that determine thermogenic capacity and WAT beiging (Figure 2). These factors include PPARγ agonists, as the master regulator of adipose tissue formation [62]. In addition, endocrine/metabolic cues such as Fibroblast Growth Factor 21 (FGF21) analogues, and AMPK activators can augment the thermogenic program and promote beiging [62–64]. FGF21 upregulates energy metabolism by stimulating BAT activation and WAT browning, and ameliorates hyperglycemia in diabetes-induced mice [65].

Lineage-specifying and beiging factors, the CCAAT/enhancer-binding protein (C/EBP) family, PR domain-containing protein 16 (PRDM16) and peroxisome proliferator–activated receptor gamma coactivator-1 alpha (PGC-1α) can trigger the beiging process of WAT [27,33,66,67]. C/EBPβ is a transcription factor that regulates the genes involved in adipogenesis and metabolism. In a previous study, C/EBPβ was shown to form a transcriptional complex with zinc finger protein PRDM16 that is sufficient to induce the transformation of myoblastic cell precursors into brown adipocytes [68]. Decreases of PRDM16, which regulates BAT development, blunt beiging by limiting the effects of PPARγ agonists [69]. Peroxisome proliferator-activated receptor gamma coactivator 1-alpha (PGC-1α) is a transcriptional coactivator that regulates the genes related to the energy metabolism. PGC-1α activates nuclear receptors and transcription factors and is involved in mitochondrial biogenesis through the glucose and lipid metabolism, oxidative phosphorylation, and adaptive response to exercise, cold and starvation. Furthermore, an increase in PGC-1α gene expression induces beiging by triggering irisin release through the upregulation of irisin precursor fibronectin type III domain-containing protein 5 (FNDC5) expression [70]. These interacting factors place PPARγ/PRDM16/C-EBPβ and PGC-1α at the core of the pro-thermogenic transcriptional module.

Among the transcriptional repressors that preserve white adipocyte identity and tune adrenergic responsiveness, KLF15, one of the 17 members of the Krüppel-like factor family, is a transcription factor with a zinc finger motif involved in various metabolic processes, including cell proliferation, differentiation, development, cardiac function, and the glucose and lipid metabolism [60]. In metabolic tissues, KLF15 restrains gluconeogenesis, modulates insulin sensitivity, and limits hepatic lipogenesis, underscoring its systemic influence. In a study by Li et al. ADRB1 was more strongly expressed in BAT when compared to subcutaneous WAT, as expected, whereas KLF15 expression was found at relatively low levels in BAT. A WAT tissue culture was exposed to the β-adrenergic stimulant isoproterenol to observe in vitro how KLF15 expression in adipose tissues responds to β-adrenergic stimuli. It was noted that KLF15 gene expression decreased in cells. When WT mice were injected with β-adrenergic agonist CL-316243, KLF15 expression in WAT was almost halved. It was concluded from these results that KLF15 is required to maintain white adipocyte properties in subcutaneous WAT, ADRB1 is a direct transcriptional target of KLF15 in white adipocytes, and KLF15 acts as a negative regulator of ADRB1 [60].

The study also showed that KLF15 in mouse adipocytes enhanced BAT properties when deleted by a Cre recombination enzyme. Brown fat markers and ADRB1 protein were upregulated in subcutaneous WAT, and WAT responded more to adrenergic stimulation, resulting in higher energy expenditures. In mice, the deletion of the KLF15 gene led to upregulated energy utilization and better temperature regulation during cold exposure, but without affecting physical activity [60]. KLF15, however, is also a key inhibitor of pathological cardiac hypertrophy, and its reduction in hypertrophied hearts and the protective effects of KLF15 overexpression, together with severe stress-induced hypertrophy in KLF15-deficient mice, highlight potential cardiac risks of systemic KLF15 suppression [71]. This duality argues for depot- and cell type–specific modulation rather than global inhibition.

Similarly, Fan et al. observed significant impairments in the lipid and amino acid metabolism in mice with BAT lacking KLF15 (K15-BKO). Both the control and K15-BKO mice were exposed to cold for 10 days to assess BAT thermogenesis, and while the K15-BKO mice were able to maintain their body temperature, like the control mice, they rapidly developed severe hypothermia during overnight fasting. A decreased expression of genes in metabolic pathways was observed that led to an imbalance in substrate utilization between BAT and other organs, such as the liver, alongside an upregulated expression of hepatic gluconeogenesis genes. These findings suggested that KLF15 is required for proper BAT function and for metabolic flexibility during cold plus fasting, and that BAT KLF15 deficiency shifts the energetic burden to the liver to compensate for impaired thermogenesis [72]. It can thus be concluded that systemic energy balance reflects coordinated cross-talk between BAT and liver when thermogenic transcriptional brakes are altered.

Zinc finger protein 423 (Zfp423), which maintains the identity of white adipocytes by repressing the thermogenic gene program, has been identified as another transcription factor that inhibits beiging in adipose tissue [73,74]. Shao et al. reported that the deletion of Zfp423 from adipose tissue in mice with metabolic syndrome impaired the terminal differentiation of white adipocytes, and subcutaneous WAT hypertrophy was subsequently observed [74]. Mechanistically, Zfp423 limits browning by suppressing early B cell factor 2 (EBF2). In this study, white adipocytes from mice with Zfp423 deleted in WAT underwent browning into beige adipocytes upon exposure to cold [75]. A recent study extending this axis demonstrated that Zfp423 deletion reactivated EBF2 in mice, upregulated adipocyte thermogenesis, and reduced obesity caused by high-fat diets consumed during the light-inactive period by enhancing phosphocreatine-creatine cycling, during which ATP synthesis takes place [76]. Given that obesity-associated insulin resistance and muscle dysfunction can reduce creatine phosphate efficiency, relieving Zfp423-mediated repression may amplify this UCP1-independent thermogenic route.

The study also evaluated brain and muscle ARNT-like 1 (BMAL1), which regulates daily biological rhythms. BMAL1 is a core clock protein that regulates the body’s circadian rhythms. It forms a complex with the CLOCK protein – a key component of the molecular circadian clock – together with BMAL1, and directs the expression of genes involved in the sleep-wake cycle, metabolism, and other daily physiological processes. In adipocytes, circadian control synchronizes thermogenic capacity with behavioral cycles, and the overexpression of BMAL1 was noted to improve metabolic outcomes in diet-induced obesity. In a previous study, changing the feeding times of mice subjected to overfeeding exacerbated metabolic syndrome, which the researchers attributed to the mismatch between adipocyte thermogenesis and the circadian rhythm [76]. These findings demonstrate that aligning feeding schedules with internal thermogenic rhythms can improve metabolic health.

Another study examining the relationship between Zfp423 and thyroid function showed how thyroid hormone affects the expression of the zinc finger transcription factor [73]. Binding of the thyroid hormone receptor to regulatory DNA near Zfp423 suppresses its transcription, whereas loss of receptor engagement upregulates Zfp423 expression. In hypothyroidism, Zfp423 expression is upregulated, leading to an increase in preadipocytes [73]. Clinically, these observations indicate a need to carefully evaluate how thyroid status reshapes adipose cellular composition and long-term metabolic risk.

In summary, adrenergic inputs, circadian timing, and thyroid signaling converge on a transcriptional core (PPARγ/PRDM16/C-EBPβ and PGC-1α) that promotes UCP1-dependent thermogenesis and the creatine phosphate cycle, while repressors (KLF15, Zfp423) maintain white adipocyte identity and tune adrenergic sensitivity. Species-specific β-adrenergic biology (human ADRB1 predominance vs rodent ADRB3 reliance) may have direct translational implications, as therapeutic strategies may prioritize ADRB1-targeted approaches and combine FGF21/AMPK-based activation with depot-restricted relief of KLF15/Zfp423 repression to safely enhance BAT/beige thermogenesis. Figure 2 clarifies these relationships.

## 4. Exosomal molecules released from adipose tissue

Exosomes are phospholipid bilayer-enclosed, cell-derived extracellular vesicles measuring 30–150 nm that contain proteins, lipids, and nucleic acids such as non-coding RNAs [77,78]. Studies over the past decade adopting differential centrifugation and complementary isolation approaches have shown that adipose-derived exosomes carry bioactive cargoes – miRNAs, lncRNAs, circRNAs, proteins, and lipids – with the ability to reprogram recipient tissues [79,80]. Collectively, these vesicles link adipose tissue to metabolic disorders and cancer by modulating the pathways that also govern brown and beige fat formation and function in both rodents and humans [81–87] (Figure 2).

miRNAs are one of the three main non-coding RNAs transported by exosomes. Over 22 nucleotides long, miRNAs recognize target mRNA with their 5’ region and bind to complementary sequences in the 3’ UTR of the mRNA, leading to mRNA degradation or the inhibition of translation. A single miRNA, which regulates nearly half of the protein-coding genes, can change the expression of up to 400 genes, while miRNA deletions or single nucleotide polymorphisms can cause changes in phenotype. lncRNAs, on the other hand, are longer than 200 nucleotides and can regulate gene expression at various levels, such as chromatin remodeling, transcriptional control, and posttranscriptional modification. A third exosomal RNA is circRNAs. These circular RNAs form covalently closed loops that are resistant to exonucleases and frequently act as miRNA “sponges”, modulating gene expression and interacting with proteins to affect cellular processes. Together, exosomal miRNAs, lncRNAs, and circRNAs create complex regulatory networks that influence adipocyte identity, thermogenesis, and whole-body energy balance [88–91].

### 4.1. tRNA fragments

In a previous study, pre-adipocytes obtained from human fetal interscapular tissue were differentiated using forskolin to identify exosomal RNAs. A distinct class of tRNA-derived fragments (tRFs) is enriched in BAT under lipolytic stimulation, with specific tRF-Gly-GCC-007, -008 and -009 increasing in BAT exposed to forskolin – a known stimulator of lipolysis. Functioning in a miRNA-like manner, tRF-Gly-GCC binds target mRNAs like UCHL1 and suppresses their expression, thereby potentially affecting lipid metabolism and storage, particularly by inhibiting BAT lipid accumulation. These tRNA fragments target and reduce the expression of ubiquitin C-terminal hydrolase L1 (UCHL1), which is involved in protein regulation and cell adhesion [87]. These findings broaden the exosomal small RNA biology beyond miRNAs, involving tRFs as modulators of BAT lipid handling.

### 4.2. circRNA

Recent studies of circRNAs have reported that some, such as circFUT10, circH19, circSMAD4A, circTshz2–1, and circArhgap5–2, regulate WAT adipogenesis, while those associated with BAT thermogenesis have been less studied [92–95]. Among the thermogenesis-relevant circRNAs, ciRS-133 (notably released by tumors), circNrxn2, and circZEB1 have emerged as regulators of browning and BAT activation. circZEB1, derived from the ZEB1 gene, sequesters miR-326–3p with a “sponge” effect and supports BAT differentiation and thermogenesis [92]. The above findings suggest that circRNAs act as exosomal rheostats in fine-tuning thermogenic gene networks, particularly in the presence of such pathophysiological conditions as cancer cachexia (see Section 4.4).

### 4.3. lncRNA

In a study examining the relationship between long non-coding RNAs and BAT, BAT-derived exosomes induced beiging in both adult stem cells and the mouse embryonic fibroblast cell line 3T3-L1, while WAT-derived exosomes did not. In a comparison with WAT-derived exosomes, BAT-derived exosomes were found to exhibit 563 differentially expressed lncRNAs, three of which (AK029592, humanlincRNA1030, and ENSMUST00000152284) showed high gene expression and were identified as potential regulators of thermogenesis and fat cell beiging [91]. These findings identify lncRNA-enriched BAT exosomes as sufficiency signals for the activation of pro-thermogenic programs in otherwise white adipocyte precursors.

### 4.4. miRNA

Exosomal miRNAs are currently the best-characterized adipose vesicle cargo in thermogenic regulation and inter-organ crosstalk. In a previous study in the literature, norepinephrine – the levels of which increase in response to cold stress – stimulated the release of miR-132-3p in the BAT exosomes of male C57/BL6J mice and affected liver lipogenesis by reducing the expression of the lipid metabolism transcription factor Srebf1 [96]. This exemplifies a BAT-to-liver axis whereby adrenergic activation exports anti-lipogenic signals via exosomes.

In a study by Lee et al., miR-375 upregulated the glycerol-3-phosphate acyltransferase (GPAM) gene in the synthesis of triglycerides and phospholipids in mouse preadipocyte cell 3T3-L1, while inhibiting leptin, which promotes lipolysis. miR-375 is also known to upregulate the expression of genes involved in fatty acid degradation, such as adipocyte differentiation (C/EBPα and PPARγ), glucose transport (GLUT4), and adiponectin receptor 2 (ADIPOR2), but while downregulating KLF2 and ADIPOQ, which encode the adipokines involved in lipid metabolism, leading to fat storage and adipocyte differentiation [97]. Although this report focused on intracellular actions, the central position of miR-375 in adipocyte lipid handling suggests its potential utility as an exosome-derived biomarker in obesity.

According to a study examining the relationship between miRNAs and exercise, the level of exosomal miR-27a (which targets the WAT master regulator PPARγ), was elevated in sedentary obese mice, but fell with training, coinciding with exercise-induced WAT beiging. Exercise also activated the IRS-1/Akt/GLUT4 signaling pathway in muscle, improving glucose metabolism and indicating its potential for insulin resistance [98]. In a similar study, experimental animals were subjected to 4 weeks of exercise, inducing an increase in BAT, although it was noted that BAT resection or silencing the BAT-derived exosome secretagogue Rab27a prior to myocardial ischemia/reperfusion reduced the beneficial effect of exercise. BAT-derived exosomal miR-125b-5p, miR-128-3p, and miR-30d-5p confer cardioprotection by coordinately suppressing the pro-apoptotic mitogen-activated protein kinase (MAPK) pathway when injected into the heart [99]. These data identify BAT exosomes as endocrine mediators of exercise benefit in multiple organs.

The first study to correlate exosomal miR-92a levels with BAT activity in humans examined the release of exosomes and their potential as biomarkers of BAT activity. The researchers used green fluorescent protein tagged to the exosome marker CD63 to detect exosomes and confirmed their release from brown adipocytes and BAT by electron microscopy and western blotting. Exposing these cells to cold-activated cAMP significantly increased exosome release, and a considerable change in miR-92a in particular. In human cohorts, lower serum exosomal miR-92a correlated with higher BAT activity on PET/CT, raising the prospect of a noninvasive biomarker to complement radiation-based imaging [86]. A similar study reported the consistent upregulation of mitochondrial folate enzyme MTHFD1L in exosomes from activated BAT (cold exposure, capsinoids, hyperthyroidism), suggesting a protein biomarker of BAT activity [100].

One of the hallmarks of cancer cachexia is WAT browning. In a study by Liu et al. (2022), exosomes released from gastric cancer cells were found to be rich in miR-155 – a specific microRNA. High levels of miR-155 in the serum and exosomes of the studied cancer patients inhibited C/EBPβ, C/EBPα, and PPARγ, leading to decreased adipogenesis and increased BAT in mesenchymal stem cells derived from patient adipose tissues. Similar results were reported in an animal model [101]. Consistent with a broader tumor-adipose axis, exosomes carrying heat shock proteins HSP70 and HSP90 aggravated cachexia by inducing lipolysis and muscle atrophy, whereas the pharmacologic blockade of exosome biogenesis (neutral sphingomyelinase inhibitor GW4869) blunted WAT browning and mitigated cachexia in vivo [102]. These observations link tumor-derived exosomes to maladaptive adipose remodeling and highlight nSMase inhibition as a potential anti-cachexia strategy.

### 4.5. Exosomal secretions

BAT-derived exosomes are gaining popularity as a cell-free therapeutic modality. For example, the weekly administration of such exosomes to mice fed a high-fat diet improved body weight, plasma glucose, glucose tolerance, and cardiac function, while in vivo delivery increased energy expenditure and improved overall metabolic health [103]. A similar study reported that the administration of BAT-derived exosomes improved oocyte maturation in vitro and in vivo by increasing the number of follicles during the estrous cycle, and supported ovarian health by enhancing mitochondrial function in oocytes [104]. Thus, BAT exosomes can convey metabolic and reproductive benefits beyond adipose tissue.

Mechanical unloading leading to increased BAT activity and higher body temperatures is a phenomenon observed in astronauts exposed to microgravity. In one notable study, bone mechanotransduction was shown to regulate an osteocyte-BAT exosome axis: gravity-like mechanical loading on bone stimulated exosomal release from osteocytes through calcium signaling, involving a miRNA let-7e-5p, which regulated BAT thermogenic activity [105]. Reduced mechanical loading (due to microgravity, bed rest, etc.) diminished osteocyte exosome output, and secondarily increased BAT activity, providing a mechanistic explanation for the increased central body temperatures observed in astronauts.

Exosomes from BAT carry inducible nitric oxide synthase (iNOS) in response to inflammatory stimuli, which can lead to cardiac fibrosis and hypertrophy. According to the study, iNOS suppression reduced the cardiac hypertrophy induced by ADRB3 antagonist SR59230A. BAT ADRB3 deletion lowered the BAT thermogenic output but increased aldosterone secretion and susceptibility to angiotensin II–induced cardiac injury, while the subsequent restoration of ADRB3 reduced exosomal iNOS and was cardioprotective [59]. These findings indicate a trade-off: while BAT activation can be beneficial, the inflammatory activation of BAT-derived exosomes can increase cardiovascular risk, necessitating context-specific modulation.

## 5. Conclusion

It can be concluded from this review that adipose-derived exosomes integrate environmental and physiological cues, including cold, exercise, mechanical stress, inflammation, and tumor burden, and transmit the ncRNAs and proteins that reshape thermogenesis, adipocyte identity, and systemic metabolism. The reviewed studies identify a core transcriptional module centered on PPARγ, PRDM16, C/EBPβ, and PGC-1α that promotes thermogenesis through UCP1 and the creatine phosphate cycle, while repressors KLF15 and Zfp423 preserve white adipocyte identity and tune adrenergic sensitivity. Species-specific β-adrenergic biology, with ADRB1 dominant in human brown fat and ADRB3 in rodents, shapes therapeutic design. Adipose-derived exosomes carry miRNAs (e.g. miR-92a, miR-132-3p, and miR-155), long noncoding RNAs, circular RNAs (such as circZEB1), tRNA fragments, and proteins (such as MTHFD1L and iNOS), and transmit signals triggered by cold, exercise, mechanical cues, inflammation, and tumors to the liver, muscles, heart, and ovaries. These vesicles offer potential as noninvasive biomarkers and cell-free therapies, but also reveal safety considerations in cases where inflammatory cargo can be harmful.
